# Personality-Related Traits and Psychological and Cognitive Outcomes Under High-Altitude Hypoxia: A Trait–Process–Outcome Framework

**DOI:** 10.3390/bs16091464

**Published:** 2026-08-24

**Authors:** Yuan Li, Shurong Jia, Tong Wu, Jinxia Cheng, Hailin Ma, Hao Li

**Affiliations:** Xizang Autonomous Region Key Laboratory for High Altitude Brain Science and Environmental Acclimatization, Xizang University, Lhasa 850000, China

**Keywords:** high-altitude hypoxia, psychological adaptation, cognitive adaptation, personality

## Abstract

High-altitude hypoxia is associated with sleep disruption, affective fluctuation, and changes in cognitive performance, yet prior findings remain markedly heterogeneous in both direction and magnitude. To clarify this variability, this structured narrative review synthesizes evidence from systematic reviews, meta-analyses, and key empirical studies on psychological and cognitive adaptation to high-altitude hypoxia, with a particular focus on personality and related traits. The review is organized along three axes: exposure context and characterization (real-altitude field/residential exposure, hypobaric chambers, normobaric hypoxia, expedition settings, and dose alignment), exposure duration and time course, and outcome domains spanning sleep/fatigue, affective adaptation, and cognitive performance. Across the literature, cognitive effects appear domain-specific and strongly conditioned by exposure dose, temporal stage, and task characteristics, whereas sleep disturbance during the early acclimatization period emerges as one of the more consistent findings and appears closely intertwined with anxiety-related responses. Although direct personality-related evidence remains limited and uneven, the available findings can be organized into a testable trait–process–outcome framework that distinguishes candidate associations with relatively more direct evidence from mechanism-informed hypotheses. Neuroticism/emotional stability and anxiety-related traits have received comparatively more direct empirical investigation than other personality domains, but the available evidence is insufficient to regard them as established predictors of high-altitude adaptation. The roles of conscientiousness, resilience/hardiness, extraversion, agreeableness, and openness remain more tentative and are better treated as targets for prospective testing. We therefore propose that personality-related dispositions may influence adaptation-relevant outcomes through candidate processes involving threat appraisal, coping, resource regulation, and social interaction. We conclude that future research should strengthen exposure dose characterization, specify the boundary conditions for generalizing across real-altitude, hypobaric-chamber, normobaric hypoxia, and expedition settings, and adopt longitudinal, multilevel, and ecologically valid designs to test personality-related pathways more rigorously.

## 1. Introduction

High-altitude hypoxia, most commonly encountered as hypobaric hypoxia, constitutes a biologically and psychologically demanding environmental stressor. Across rapid ascent, short-term deployment, and longer-term residence, exposure to high altitude can alter sleep, affective experience, and cognitive performance, thereby influencing both subjective well-being and functional efficiency (Kious et al., 2018; Roach et al., 2018; Su et al., 2024). Among these outcomes, sleep disturbance is one of the earliest and most frequently reported complaints, whereas cognitive effects appear to involve multiple domains, including attention, executive control, and memory (Bliemsrieder et al., 2022; Bloch et al., 2015). At the same time, the magnitude and even direction of observed effects vary substantially across studies, suggesting that adaptation to high-altitude hypoxia cannot be adequately understood in terms of uniform mean effects alone and instead requires closer attention to individual differences and study boundary conditions.

Beyond somatic discomfort and broad behavioral changes, psychological adaptation to high-altitude hypoxia has also attracted increasing attention. Population-level evidence suggests that residence at, or migration to, higher altitude may be associated with elevated risk of depressive and anxiety symptoms, although such associations require cautious interpretation in light of potential confounding by demographic composition, socioeconomic context, healthcare accessibility, and migration-related selection effects (Basualdo-Meléndez et al., 2022; Kious et al., 2019). At a more proximal level, one of the most informative patterns may lie in the coupling between sleep disruption and affective responses during the early phase of exposure. In a field study conducted at 3700 m, acute high-altitude exposure significantly increased the incidence of sleep disturbance, which was closely associated with anxiety levels; baseline anxiety and age further predicted subsequent sleep problems (Bian et al., 2019). More broadly, reviews suggest that hypoxic stress and psychological stress may interact through partially overlapping pathways, including inflammation, autonomic activation, and altered regulatory signaling, thereby providing a plausible context in which individuals exposed to comparable hypoxic loads may nevertheless show markedly different affective and cognitive trajectories (Burtscher et al., 2022).

A central implication of the heterogeneity reviewed above is that adaptation to high-altitude hypoxia cannot be understood solely in terms of exposure intensity or average group-level effects. Even at the same altitude and under broadly comparable exposure duration and task demands, individuals may differ substantially in affective reactivity, sleep disruption, symptom reporting, and cognitive stability. Such variability is likely to reflect not only differences in study design and task selection, but also enduring person-level characteristics that shape how hypoxic stress is perceived, regulated, and behaviorally managed. In this context, personality and related trait constructs—such as neuroticism/emotional stability, trait anxiety, resilience/hardiness, and coping-related dispositions—provide a promising and theoretically tractable entry point. These traits may influence adaptation by biasing threat appraisal, modulating affective arousal, shaping coping-strategy selection, and altering the allocation of self-regulatory resources, with downstream consequences for sleep-related recovery, subjective symptom burden, and the maintenance of cognitive performance under task load. Available trait-relevant findings from extreme-hypoxia and confined-environment research suggest that such a perspective is empirically plausible and worthy of more systematic investigation in real-world high-altitude contexts (Nicolas et al., 1999, 2000).

Against this background, the present article provides a structured narrative review of psychological and cognitive adaptation to high-altitude hypoxia, with a particular emphasis on personality and related traits as potential sources of individual variability. Our primary aim is not to claim that personality effects have already been firmly established across high-altitude settings, but rather to evaluate the current evidence base, identify where direct empirical evidence is relatively more developed, and clarify where the literature remains indirect, fragmented, or largely mechanism-informed. To this end, we prioritize systematic reviews, meta-analyses, and empirical studies conducted in high-altitude or hypoxia-related contexts, while drawing more selectively on adjacent evidence from extreme-environment and stress research when it helps illuminate plausible trait-related pathways. The review is organized around four questions: how high-altitude hypoxia affects psychological and cognitive outcomes; why findings vary as a function of exposure characterization, time course, and task demands; which personality and related traits appear most relevant to adaptation; and what kinds of longitudinal, multilevel, and ecologically valid designs are needed to test these pathways more rigorously in future work.

## 2. Methods

### 2.1. Structured Narrative Review Approach

This article was conducted as a structured narrative review designed to balance conceptual integration with methodological transparency. Unlike a full systematic review, the present review was not intended to exhaustively enumerate every published study or to perform a formal quantitative synthesis. Rather, its purpose was to organize and interpret the available literature in a theory-informed and evidence-sensitive manner while making the search strategy, eligibility boundaries, source-selection logic, and evidence hierarchy explicit.

The review followed core quality principles for rigorous narrative reviews, including clarity of scope, transparency of literature identification and selection, and explicit differentiation between stronger and more tentative forms of evidence (Baethge et al., 2019; Ferrari, 2015; Gasparyan et al., 2011; Sukhera, 2022). Relevant principles from systematic-review reporting guidance were also used selectively to improve transparency in relation to information sources, search reproducibility, eligibility criteria, and documentation of record retrieval, without claiming compliance with the methodological requirements of a full systematic review (Page et al., 2021).

### 2.2. Information Sources and Search Strategy

Literature searches were conducted in PubMed and Web of Science. The initial PubMed search was conducted on 19 December 2025, and the initial Web of Science search was conducted on 26 December 2025. Only English-language publications were considered, and no lower publication-date restriction was applied.

Two complementary search modules were used to reflect the dual scope of the review. Search A was designed to identify review-level evidence concerning psychological and cognitive consequences of high-altitude or hypoxic exposure. Exposure-related terms were combined with terms representing sleep/fatigue, affective outcomes, and cognitive performance, together with terms identifying reviews and meta-analyses. Search B was designed to identify evidence concerning personality and related individual-difference factors in high-altitude or hypoxic adaptation. Exposure-related terms were therefore combined with personality- and trait-related terms and with adaptation-relevant psychological, behavioral, symptom, and cognitive outcomes.

Search A retrieved 304 records from PubMed and 307 records from Web of Science. Search B retrieved 212 records from PubMed and 489 records from Web of Science. Thus, the four search runs yielded 1312 records before de-duplication. Records from both databases and both search modules were subsequently merged, and duplicate records were identified using bibliographic identifiers, including DOI and PMID where available, supplemented by title-based matching. A total of 341 duplicate records were removed, leaving 971 unique records in the updated search pool.

Database searching was supplemented by backward citation searching of relevant systematic reviews, meta-analyses, and key empirical studies. Citation tracing was used purposively to identify conceptually important publications that were not retrieved by the principal database searches, particularly in adjacent literatures concerning extreme environments, personality, stress adaptation, and confined or hypoxic settings (Bliemsrieder et al., 2022; McMorris et al., 2017; Ramírez-delaCruz et al., 2024). Because citation tracing served as a supplementary narrative-review procedure rather than a separately logged exhaustive search stream, a separate historical retrieval count was not retained.

The complete database-specific search strategies, language restriction, and retrieval counts are provided in Table 1.

### 2.3. Eligibility Criteria, Screening, and Source Selection

Sources were considered eligible for the substantive evidence synthesis when they involved adult human participants exposed to real high altitude or simulated hypoxic conditions, including hypobaric and/or normobaric paradigms, and addressed outcomes relevant to psychological or cognitive functioning under hypoxic exposure. Relevant outcomes included anxiety, depressive symptoms, mood or affective responses, stress, sleep disturbance, fatigue, attention, executive function, memory, broader cognitive performance, acute mountain sickness–related symptom burden, or closely related functional indicators.

For the trait-focused component of the review, priority was given to studies assessing broad personality dimensions, including Big Five traits, or theoretically adjacent individual-difference constructs with potential relevance to high-altitude outcomes, including trait anxiety, resilience/hardiness, coping-related dispositions, and sensation seeking. Studies were excluded from the substantive synthesis when they focused exclusively on physiological acclimatization without a meaningful psychological, behavioral, symptom-related, or cognitive outcome. Pediatric studies, non-human studies, and hypoxia paradigms without meaningful relevance to high-altitude exposure were also excluded. The adequacy of hypoxic exposure characterization was considered in interpreting the comparability and directness of evidence using the multidimensional exposure dose definition provided in Section 3.2; it was not used as a formal risk-of-bias or study-quality score.

Across the finalized database searches, 1312 records were identified before deduplication: 304 from PubMed Search A, 212 from PubMed Search B, 307 from Web of Science Search A, and 489 from Web of Science Search B. Removal of 341 duplicate records yielded 971 unique records for title- and abstract-level relevance assessment. Potentially relevant sources identified at this stage were subsequently considered in greater detail against the predefined eligibility criteria. Because the review followed an evidence-prioritization approach rather than a prospectively documented systematic-review screening workflow, the exact number of records progressing to detailed consideration was not retained and was not reconstructed retrospectively. Screening and source selection were conducted by the first and second authors, and the second author additionally verified the final eligibility decisions. Uncertainties regarding relevance or eligibility were resolved through discussion between the two authors.

Source selection followed an evidence-prioritization rather than strict exhaustiveness principle, consistent with the structured narrative design. Systematic reviews and meta-analyses were preferentially used to anchor broader conclusions regarding psychological and cognitive outcomes under high-altitude hypoxia. Primary empirical studies conducted directly under real-altitude or experimentally controlled hypoxic conditions were prioritized when evaluating personality- and trait-related associations. Evidence from mountaineering, adjacent extreme-environment, and broader stress or personality literatures was incorporated more selectively when it contributed contextual or mechanism-informed support relevant to the trait–process–outcome framework. Evidence from these adjacent literatures was not treated as equivalent to direct high-altitude or experimental-hypoxia evidence.

The final manuscript cites 51 sources in total. This number represents the complete set of sources used across the manuscript rather than 51 database-derived empirical studies. The cited literature includes substantive empirical studies, systematic reviews and meta-analyses used in the evidence synthesis, as well as theoretical, methodological, and measurement references used to define constructs, develop the conceptual framework, and illustrate future assessment approaches. Accordingly, the total reference count should be interpreted as a final cited-source count rather than as a conventional systematic-review count of included primary studies.

### 2.4. Synthesis Framework and Evidence-Tiering Criteria

The literature was synthesized along three principal organizing axes: exposure characterization, exposure duration, and outcome domain. Exposure characterization distinguished real high-altitude exposure from simulated hypoxic paradigms and considered the degree to which the hypoxic dose was characterized. Exposure duration broadly differentiated acute or short-term entry phases from longer-term residence or adaptation phases. Outcome domains were organized into sleep/fatigue, affective adaptation, symptom or functional burden, and cognitive performance, while recognizing that these domains may interact.

To distinguish between relatively well-supported associations and more provisional interpretations, evidence strength was evaluated qualitatively across six dimensions. First, amount of evidence referred to the number of relevant studies and whether systematic reviews or meta-analyses were available. Second, study design considered whether evidence derived from longitudinal or repeated-measures studies, controlled hypoxia experiments, well-characterized field studies, or primarily cross-sectional and correlational designs. Third, directness of evidence referred to whether the trait–adaptation association was tested directly during real high-altitude or experimentally controlled hypoxic exposure, as opposed to being inferred from adjacent extreme-environment, personality, or general stress research. Fourth, replication referred to whether conceptually comparable associations had been observed across independent samples or exposure settings. Fifth, measurement quality considered the use of validated personality or psychological measures, standardized cognitive or functional outcomes, and adequate characterization of hypoxic exposure (e.g., altitude, exposure duration, or physiological indices where reported). Sixth, consistency referred to the degree of convergence in the direction and interpretation of findings across the available evidence.

Based on these dimensions, four qualitative evidence categories were used (Table 2). Stronger/convergent evidence was assigned when an association was supported by multiple direct high-altitude or hypoxia studies, replicated across independent samples or settings, and/or reinforced by review-level evidence, with generally consistent findings and appropriate measurement. Moderate or emerging direct evidence referred to associations supported by at least one directly relevant high-altitude or hypoxia study, or a small number of reasonably consistent direct studies, but for which replication, design strength, or measurement coverage remained limited. Limited/indirect evidence was used when direct high-altitude evidence was sparse or inconsistent and the interpretation depended substantially on adjacent extreme-environment findings or indirect process evidence. Finally, a mechanism-informed hypothesis denoted a theoretically plausible pathway supported primarily by established personality, stress, coping, resource-regulation, or person–environment theories, but not yet directly or consistently demonstrated in high-altitude populations.

These categories were not generated through a numerical scoring algorithm, and no single dimension automatically determined the classification. Rather, the six dimensions were considered jointly, with particular emphasis on directness, replication, measurement quality, and consistency. Accordingly, a large number of indirect or methodologically heterogeneous studies was not considered equivalent to replicated, directly relevant high-altitude evidence. Likewise, a single well-designed study could provide important direct support but was not, by itself, considered sufficient for the strongest evidence category.

The evidence-tiering procedure was intended to structure interpretation and prevent overstatement rather than to constitute a formal risk-of-bias or certainty-of-evidence assessment. No formal risk-of-bias or GRADE-type evaluation was undertaken because the objective of the present article was structured narrative synthesis rather than systematic evidence grading or quantitative meta-analysis.

Exposure context and exposure duration were also treated as explicit dimensions of evidence directness. For conclusions concerning real-world high-altitude adaptation, longitudinal or repeated-measures evidence obtained during actual high-altitude exposure was considered more directly applicable than evidence obtained exclusively from short-duration simulated hypoxia. Hypobaric-chamber and normobaric hypoxia studies were used primarily to support experimentally controlled or mechanism-oriented interpretations, with the two paradigms distinguished rather than pooled automatically. Mountaineering and expedition studies were treated as ecologically relevant but potentially influenced by physical, environmental, social, and sample-selection factors beyond hypoxia itself. Evidence from adjacent extreme environments was considered indirect and was used for mechanistic interpretation or hypothesis generation rather than as direct evidence of high-altitude adaptation. Generalization across these contexts therefore required convergence in exposure characteristics, temporal phase, outcome domain, and direction of findings.

## 3. Conceptual Scope and Definitions

### 3.1. Scope of the Review Question

This review addresses a focused question: under conditions of high-altitude hypoxic exposure, which personality dimensions and related traits are most relevant to individual differences in psychological and cognitive adaptation, through what kinds of psychological–behavioral processes might these associations operate, and under which exposure and study conditions are such associations more or less likely to emerge. Because the literature is marked by conceptual and measurement heterogeneity, we define the central constructs of exposure, outcomes, and trait variables before proceeding to the substantive synthesis. This step is intended to improve interpretive consistency and to reduce ambiguity in how findings from partially overlapping literatures are compared and integrated.

### 3.2. Exposure-Context Classification and Generalizability

The evidence considered in this review originates from several exposure contexts that differ in ecological validity, experimental control, exposure duration, and accompanying environmental or psychosocial demands. To avoid treating these contexts as interchangeable, we distinguish five broad categories: real high-altitude field or residential exposure, hypobaric-chamber simulation, normobaric hypoxia, mountaineering or expedition settings, and adjacent extreme-environment research.

For consistency throughout this review, hypoxic exposure dose refers to the combined magnitude and duration of the environmental hypoxic stimulus together with the physiological hypoxemia experienced by participants. Environmental exposure may be characterized by nominal altitude, inspired oxygen fraction (FiO_2_), and/or inspired oxygen partial pressure (PIO_2_), whereas temporal characterization includes exposure duration and exposure phase. Where available, physiological indicators such as mean SpO_2_, nadir SpO_2_, and nocturnal oxygenation variability provide complementary information about the realized hypoxic burden. No single metric is assumed to capture hypoxic exposure dose fully; rather, these indicators are considered jointly when evaluating the comparability and interpretability of findings across studies.

Real high-altitude field or residential exposure refers to participants physically present at altitude under naturally reduced barometric pressure, including both acute entry and longer-term residence. These studies provide the most ecologically direct evidence for outcomes occurring in actual high-altitude environments, but they also permit greater variation in workload, sleep opportunity, environmental conditions, prior acclimatization, and social context. For questions concerning prolonged exposure, longitudinal or longer-duration real-altitude studies were therefore considered more directly applicable than short experimental protocols.

Hypobaric-chamber studies reproduce reduced barometric pressure under controlled conditions and provide experimentally controlled evidence concerning hypobaric hypoxia. However, chamber exposure does not reproduce the full ecological, behavioral, occupational, and social context of real-altitude environments. Such studies were therefore treated as direct evidence for responses to controlled hypobaric exposure, but not as automatically equivalent to real-world high-altitude outcomes.

Normobaric hypoxia studies reduce inspired oxygen concentration without reducing barometric pressure and provide strong experimental control for examining hypoxia-related mechanisms and short-term psychological or cognitive responses. Nevertheless, normobaric and hypobaric hypoxia should not be regarded as physiologically or functionally interchangeable, because differences have been reported in physiological responses and cognitive performance even under broadly comparable inspired oxygen partial-pressure conditions (Hohenauer et al., 2022). Findings from normobaric protocols were therefore generalized beyond the experimental setting only when the exposure dose, duration, and research question were sufficiently comparable.

Mountaineering and expedition studies were treated as a distinct form of real-altitude field evidence because altitude exposure is typically embedded within sustained physical exertion, disrupted recovery, environmental adversity, operational constraints, and social or team demands. These samples may also be subject to self-selection, with mountaineers and expedition members differing in personality characteristics from less selected populations (Crust, 2020; Monasterio et al., 2014). Findings from such settings were therefore interpreted in relation to their specific ecological and sampling characteristics rather than generalized automatically to people living or working at altitude.

Finally, adjacent extreme-environment research includes evidence from demanding, isolated, or confined environments that may inform candidate processes such as coping, resource regulation, social adaptation, or stress appraisal but does not provide direct evidence of hypoxia-related effects. Such studies were used for theoretical interpretation and hypothesis generation rather than as direct evidence for high-altitude or experimentally induced hypoxic outcomes.

Exposure context was considered jointly with exposure phase. We distinguish broadly between acute or entry-phase exposure, typically spanning hours to several days, and mid- to long-term exposure, typically spanning weeks or longer. Acute exposure is more informative for outcomes such as symptom burden, sleep disruption, and affective fluctuation, whereas prolonged exposure permits evaluation of maintenance, recovery, and longer-term psychological or cognitive functioning under sustained demands (Bloch et al., 2015; Nicolas et al., 1999, 2000; Roach et al., 2018). Accordingly, findings from short-duration simulated hypoxia were not interpreted as direct evidence for prolonged real-altitude outcomes unless supported by convergent longitudinal or field evidence.

### 3.3. Outcome Domains and Analytical Boundaries

In high-altitude research, the term adaptation is used broadly and may refer to changes in symptom burden, sleep and fatigue, affective or mental-health status, cognitive performance, or broader functional outcomes. In the present review, adaptation is therefore used as an umbrella term for multiple analytically distinct domains rather than as a single unitary construct or a fixed sequential process. Improvement or impairment in one domain is not assumed to imply corresponding change in another.

The first domain comprises acute symptoms and functional status, including acute mountain sickness (AMS)–related symptoms and associated functional disruption, with the Lake Louise 2018 framework providing a useful reference for standardized assessment during early exposure (Roach et al., 2018). The second domain comprises sleep and fatigue, including subjective sleep quality, insomnia-related symptoms, daytime sleepiness, fatigue, and, where available, objective sleep indices. The third domain comprises affective and mental-health outcomes, including anxiety, depressive symptoms, stress, and affective instability. The fourth domain comprises cognitive performance, including attention, executive control, memory, and related performance indices. Broader social or occupational functioning may provide an additional ecological outcome domain in prolonged or operational settings, but such outcomes should not be treated as interchangeable with psychological symptoms or cognitive performance.

These domains may covary or influence one another, but the available evidence does not establish a single sequence through which high-altitude adaptation necessarily unfolds. For example, sleep disturbance may coexist with anxiety or cognitive difficulty without demonstrating that one mediates the other. Similarly, AMS symptom burden should not be interpreted as a proxy for affective or cognitive maladaptation.

Sleep and fatigue are therefore treated as cross-cutting variables whose analytical role depends on the research question. They may be outcomes of hypoxic exposure in their own right, but in longitudinal models they may also be evaluated as candidate intermediate variables or potential mediators linking exposure or person-level characteristics to later affective or cognitive outcomes. Such mediating roles are considered hypotheses unless mediation has been tested directly (Bian et al., 2019; Bloch et al., 2015; Burtscher et al., 2022).

### 3.4. Personality and Related Traits: Conceptual Boundaries

The Big Five model serves as the primary organizing framework for broad personality dispositions in this review, given its established measurement tradition, relative comparability across studies, and relevance to affective, behavioral, and self-regulatory functioning (John & Srivastava, 1999). Within this framework, neuroticism refers to a broad dispositional tendency toward negative affectivity, emotional reactivity, and sensitivity to stress, whereas emotional stability is treated as the inverse pole of the same personality dimension rather than as a separate construct. Extraversion, conscientiousness, agreeableness, and openness are likewise treated as broad personality dimensions whose relevance to high-altitude adaptation is considered according to the strength and directness of the available evidence.

Several narrower trait-like constructs are included because they may provide additional explanatory value, but they are not treated as interchangeable with the Big Five. Trait anxiety is conceptually related to neuroticism but is narrower in scope, referring to a relatively stable disposition to perceive or respond to situations as anxiety provoking (Spielberger et al., 1983). Anxiety sensitivity is treated as a more specific vulnerability involving heightened concern about anxiety-related or bodily sensations and their perceived consequences. Although these constructs may overlap empirically with neuroticism, evidence involving trait anxiety or anxiety sensitivity is not considered direct evidence for neuroticism unless neuroticism itself was explicitly measured. This distinction is particularly important in the high-altitude literature, where anxiety-related measures have sometimes been examined alongside personality and altitude-related symptomatology (Nicolas et al., 1999, 2000).

Resilience and hardiness are also treated as related but non-equivalent constructs. Resilience refers here to the capacity or dispositional tendency to maintain or recover psychological functioning in the face of adversity (Connor & Davidson, 2003), whereas hardiness refers more specifically to a dispositional orientation characterized by commitment, perceived control, and the tendency to approach demanding circumstances as challenges rather than solely as threats (Kobasa, 1979). Both constructs may be relevant to adaptation under sustained environmental stress, but neither is treated as equivalent to a Big Five trait or to the other.

Coping style and self-regulation occupy a different conceptual level. Coping style refers to relatively habitual patterns of responding to stress, whereas specific coping strategies may vary across situations (Carver, 1997; Carver & Connor-Smith, 2009). Self-regulation refers more broadly to the processes through which individuals manage behavior, effort, attention, emotion, and resource allocation in pursuit of goals. In the present trait–process–outcome framework, coping and self-regulation are therefore treated primarily as intermediate psychological or behavioral processes, or as process-related dispositions, rather than as broad personality traits. Sensation seeking and risk preference are included more selectively as narrower individual-difference constructs when they provide specific explanatory value for risk-related behavior in high-altitude or adjacent extreme-environment settings (Zuckerman, 1994).

Finally, the review distinguishes these relatively stable dispositional constructs from transient states and adaptation outcomes. State anxiety, acute distress, sleep disturbance, fatigue, and cognitive performance are treated as context-sensitive responses, processes, or outcomes rather than as indicators of broad personality traits. Accordingly, an association between high-altitude exposure and anxiety or sleep disturbance does not by itself constitute evidence that neuroticism predicts adaptation. Such findings are attributed to neuroticism only when neuroticism is measured directly; otherwise, they are interpreted at the level of the construct actually assessed or used to motivate an explicitly theory-informed pathway. This distinction is maintained throughout the review to reduce construct conflation and to ensure that evidence is interpreted at the level at which it was measured (Burtscher et al., 2022; Carver & Connor-Smith, 2009; Nicolas et al., 1999, 2000).

## 4. A Trait–Process–Outcome Framework for High-Altitude Adaptation

**Figure 1:**
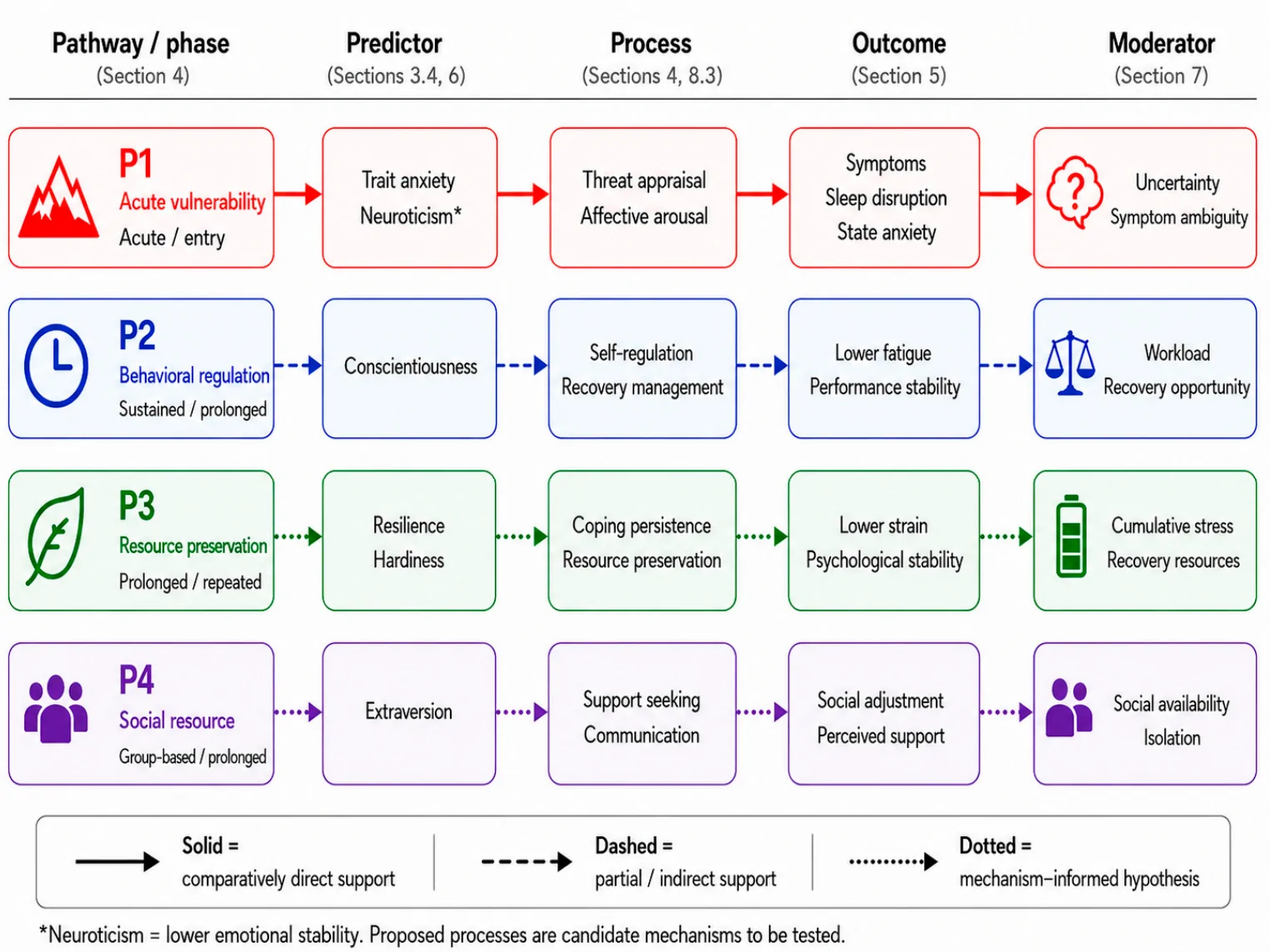
Trait–process–outcome framework for psychological and cognitive outcomes under high-altitude hypoxia.

To integrate the heterogeneous literature reviewed here, we propose a trait–process–outcome framework that distinguishes distal dispositional variables, candidate psychological or behavioral processes, and domain-specific adaptation outcomes (Figure 1). The framework does not assume that every personality trait influences every process or outcome. Instead, it identifies a limited set of falsifiable pathways in which broad personality traits or narrower trait-like constructs are specified as candidate predictors, coping and self-regulatory variables may operate as intermediate processes, and symptom, affective, cognitive, or functional outcomes are examined separately according to the specific hypothesis.

The framework draws on complementary theoretical traditions. Transactional stress theory informs pathways involving threat appraisal and coping (Lazarus & Folkman, 1984; Carver & Connor-Smith, 2009), whereas Conservation of Resources theory informs hypotheses concerning cumulative fatigue, recovery, and preservation of regulatory resources during sustained exposure (Hobfoll, 1989). Trait activation theory provides a basis for expecting trait effects to vary with situational demands (Tett & Burnett, 2003), while person–environment fit highlights the potential importance of alignment between dispositional tendencies and environmental or social requirements (Kristof, 1996). These theories are used to generate testable mechanisms rather than as substitutes for direct high-altitude evidence.

Four focal propositions are prioritized below. Each specifies a predictor, candidate intermediate process, outcome, expected direction, exposure phase, and principal boundary condition. The propositions differ in evidential status: some component associations have direct high-altitude or hypoxia support, whereas others remain primarily indirect or mechanism-informed.

**Proposition 1.**  *Acute threat–arousal pathway.*

During acute or entry-phase high-altitude/hypoxic exposure, higher trait anxiety is hypothesized to be associated with greater acute psychological or symptom-related burden, with heightened threat appraisal and affective arousal as candidate intermediate processes. Where neuroticism is measured directly, higher neuroticism/lower emotional stability is expected to show a similar association with affective or symptom vulnerability; however, neuroticism and trait anxiety should be analyzed as distinct predictors rather than treated as interchangeable constructs. These associations may be more detectable under conditions of high uncertainty, ambiguous bodily sensations, and disrupted recovery. Available field and hypobaric-chamber studies provide some direct empirical observations concerning selected anxiety- and personality-related associations, but this evidence remains sparse, construct-specific, and insufficiently replicated; the full mediated sequence remains untested (Bian et al., 2019; Burtscher et al., 2022; Nicolas et al., 1999, 2000).

**Proposition 2.**  *Behavioral self-regulation and recovery pathway.*

During sustained or prolonged high-altitude exposure, higher conscientiousness is hypothesized to be associated with better maintenance of functioning through behavioral self-regulation and recovery management. Candidate processes include pacing, adherence to recovery routines, effort regulation, and management of sleep and fatigue. The expected outcome is greater stability of cognitive or operational functioning and lower cumulative fatigue rather than uniformly superior performance at a single assessment. This association is expected to depend on workload and recovery opportunity: persistence may be advantageous when adequate recovery is available but less beneficial when sustained demands promote continued effort despite insufficient restoration. This pathway remains primarily mechanism-informed rather than directly established in high-altitude populations (Carver & Connor-Smith, 2009; Hobfoll, 1989; Bliemsrieder et al., 2022; Fan et al., 2024).

**Proposition 3.**  *Resource-preservation pathway.*

During prolonged or repeated high-altitude exposure, higher resilience or hardiness is hypothesized to be associated with better maintenance or recovery of psychological functioning through adaptive coping and preservation of psychological resources. Expected outcomes include lower cumulative affective strain or fatigue and greater stability of psychological functioning over time. These associations may become more detectable as cumulative demands increase and are expected to depend on the availability of recovery resources. Resilience and hardiness should be measured as related but distinct constructs, and their proposed roles through coping persistence or resource preservation remain primarily theory informed and require direct longitudinal testing in high-altitude settings (Carver & Connor-Smith, 2009; Hobfoll, 1989; Kobasa, 1979).

**Proposition 4.**  *Social-resource pathway.*

In prolonged high-altitude settings involving group living or coordinated work, higher extraversion is hypothesized to be associated with better affective or social adjustment when opportunities for interpersonal interaction and support are available. Social-support seeking and communication represent candidate intermediate processes. The expected direction is conditional rather than uniformly positive: extraversion may be advantageous in socially interactive environments, whereas its association with adjustment may be attenuated or potentially reversed under isolation or restricted social contact. This proposition is supported mainly by broader personality, coping, and person–environment perspectives and remains limited or indirect in the high-altitude literature (Burtscher et al., 2022; Carver & Connor-Smith, 2009; Kristof, 1996; Tett & Burnett, 2003).

These four propositions convert the broader framework into specific research targets without implying that the proposed mediating mechanisms are established. Proposition 1 currently has comparatively more direct empirical anchors within the limited literature, whereas Propositions 2–4 depend increasingly on indirect evidence and theory-informed inference. Testing these pathways requires designs that measure the predictor, candidate intermediate variable, outcome, exposure phase, and relevant moderating condition within the same study.

Finally, the outcome categories in the framework should be interpreted as analytically distinct domains rather than stages of a common adaptation sequence. Symptom burden, sleep/fatigue, affective outcomes, cognitive performance, and social or occupational functioning may covary but should not be assumed to form a fixed causal progression. Likewise, when sleep, fatigue, affective regulation, coping, or self-regulation is positioned between a trait and an outcome, it represents a candidate mediation hypothesis requiring direct temporal and statistical testing, not an established mechanism.

## 5. Overall Effects of High-Altitude Hypoxia on Psychological and Cognitive Outcomes

### 5.1. Symptoms and Functional Status

During the acute entry phase at high altitude, one of the most common indicators of impaired adaptation is acute mountain sickness (AMS), typically characterized by headache, gastrointestinal discomfort, fatigue or weakness, and dizziness (Roach et al., 2018). The Lake Louise 2018 scoring system provides a relatively standardized framework for quantifying this early symptom burden and thus offers a useful benchmark for comparing functional impairment across studies (Roach et al., 2018). At the same time, AMS should be interpreted primarily as an index of symptom burden and functional maladaptation during early exposure, rather than as a direct proxy for either mental-health outcomes or cognitive performance. This distinction is important because subjective symptom severity may covary with affective reactivity, sleep disruption, and interoceptive sensitivity without mapping cleanly onto downstream cognitive impairment.

### 5.2. Sleep and Fatigue

Among the psychological and behavioral consequences of acute high-altitude exposure, sleep disturbance is one of the most frequent and comparatively consistent findings. In low-altitude individuals ascending to high altitude, sleep problems often emerge within the first few nights and are linked to hypoxia-related ventilatory instability, sleep fragmentation, and reductions in restorative sleep architecture (Bloch et al., 2015). For the purposes of the present review, sleep and fatigue are treated as distinct adaptation-relevant outcomes that may also function as candidate intermediate variables in specific longitudinal models. Sleep disturbance and fatigue can plausibly contribute to subsequent affective or cognitive variability, but their mediating roles should not be assumed in the absence of direct temporal or mediation analyses. Accordingly, both subjective indices (e.g., sleep quality, insomnia symptoms, daytime sleepiness) and objective indicators (e.g., PSG- or wearable-derived sleep measures, where available) should be measured explicitly when these pathways are tested (Bloch et al., 2015).

Sleep disturbance during the entry phase also appears closely associated with affective responses. In a field study conducted at 3700 m, acute high-altitude exposure increased the incidence of sleep disturbance, which was associated with anxiety levels; baseline anxiety and age further predicted subsequent sleep problems (Bian et al., 2019). This finding provides direct evidence for an association between anxiety and sleep disturbance during early high-altitude exposure, but it does not establish a bidirectional or mediating relationship. More broadly, review-level evidence suggests that hypoxic and psychological stress may interact across multiple regulatory systems, providing a plausible context for correlated sleep and affective disturbances during adaptation (Burtscher et al., 2022). Sleep and anxiety should therefore be treated as closely related but analytically distinct outcomes or candidate intermediate variables whose temporal relationships require prospective testing.

### 5.3. Affect and Mental Health

Population-level evidence suggests that residence at, or migration to, higher altitude may be associated with elevated depressive and anxiety symptoms. A systematic review and meta-analysis estimated a pooled prevalence of depressive symptoms of 17.9% among high-altitude residents, with a country-level estimate of 28.7% for China (Basualdo-Meléndez et al., 2022). However, the overall estimate was highly heterogeneous, and these associations should be interpreted cautiously because demographic composition, socioeconomic conditions, healthcare accessibility, measurement approaches, and migration-related selection may contribute to the observed patterns (Basualdo-Meléndez et al., 2022; Kious et al., 2019).

Acute anxiety-related responses, including their association with sleep disturbance, are summarized separately in Section 5.2 and should not be conflated with longer-term mental-health outcomes. More broadly, review-level evidence suggests that hypoxic and psychological stress may interact through partially overlapping inflammatory, autonomic, and regulatory processes, although these pathways remain incompletely established in high-altitude populations (Burtscher et al., 2022). Such evidence provides a plausible context for studying individual differences in affective responses but does not establish specific causal or mediating mechanisms.

### 5.4. Cognitive Outcomes

Cognitive effects of high-altitude hypoxia are heterogeneous (Bahrke & Shukitt-Hale, 1993), but recent evidence indicates that this heterogeneity should not be interpreted as an absence of an overall adverse effect. Bliemsrieder et al. (2022), in a systematic review of 52 studies using 112 different neuropsychological tests, found substantial variation between laboratory and field settings. In laboratory studies, approximately 64.7% of reported test results indicated deterioration, whereas in field studies 66.4% indicated either no change or improvement. The authors suggested that greater acclimatization in field settings may partly contribute to this contrast. The review also identified attention/concentration and executive functions as the most frequently investigated domains, while highlighting substantial variation in the sensitivity of individual tasks and paradigms (Bliemsrieder et al., 2022). Fan et al. (2024) similarly documented pronounced methodological heterogeneity, identifying 107 distinct cognitive tests and showing that attention/concentration, memory, and executive function have received the greatest research attention. Because this review focused primarily on assessment methods rather than pooled effects, its main contribution is to demonstrate the difficulty of comparing nominally similar cognitive domains across studies.

Quantitative syntheses nevertheless provide clearer evidence regarding direction and magnitude. In a meta-analysis of 49 studies involving 6191 participants, Su et al. (2024) found that long-term high-altitude exposure was associated with an overall moderate reduction in cognitive performance (Hedges’ *g* = −0.40, 95% CI [−0.76, −0.05]). The effects were selective rather than global: psychomotor function and long-term memory showed the most pronounced deterioration, working memory and language showed more moderate reductions, whereas perceptual processes, inhibitory control, and problem-solving did not show significant impairment. Cognitive impairment was also more evident at altitudes above 4000 m and among high-altitude immigrants than in better-adapted resident populations (Su et al., 2024).

A broader three-level meta-analysis by Jiang et al. (2025), comprising 59 studies and 739 effect sizes across different exposure durations, similarly identified an overall detrimental effect of high-altitude hypoxia on cognition (*g* = −0.424). However, the magnitude differed substantially across domains: perceptual ability (*g* = −0.923) and long-term memory (*g* = −0.572) showed the largest pooled effects, followed by attention (*g* = −0.422), executive control (*g* = −0.403), psychomotor function (*g* = −0.353), and working memory (*g* = −0.322). Effects were significant at altitudes above 2500 m and were evident during acute (<3 days) and longer-term (>30 days) exposure, whereas intermediate exposure of 3–30 days did not show a significant overall effect (Jiang et al., 2025).

Taken together, these findings support a selective and exposure-dependent rather than uniform model of cognitive impairment. Long-term memory emerges as a comparatively consistent vulnerable domain across quantitative syntheses, while psychomotor performance also appears particularly susceptible during prolonged exposure. By contrast, findings for working memory, executive control, attention, and especially perceptual functions vary according to exposure duration, altitude, study context, and cognitive classification. For example, perceptual processes were not significantly impaired in the long-term meta-analysis by Su et al. (2024), whereas Jiang et al. (2025), which integrated a broader range of exposure durations and paradigms, reported a comparatively large pooled perceptual effect. Such differences reinforce the need to interpret cognitive outcomes in relation to exposure phase and measurement structure rather than assuming that hypoxia produces a single global pattern of impairment (Bliemsrieder et al., 2022; Fan et al., 2024; Jiang et al., 2025; Su et al., 2024).

## 6. Personality and Related Traits as Sources of Individual Differences in High-Altitude Adaptation

The available literature suggests that personality and related traits may contribute to individual differences in high-altitude adaptation-related outcomes, but the empirical evidence remains limited and uneven across trait domains. Accordingly, this section distinguishes associations examined directly under real-altitude or experimentally controlled hypoxic exposure from pathways supported primarily by broader personality, stress, coping, or person–environment research.

Within this limited evidence base, neuroticism/emotional stability and anxiety-related characteristics have received comparatively more direct empirical investigation than other trait domains, although the available findings derive from only a small number of field and controlled-hypoxia studies and remain heterogeneous across constructs and outcomes (Bian et al., 2019; Nicolas et al., 1999, 2000; Burtscher et al., 2022). This relative difference in evidential coverage does not establish these constructs as reliable predictors of high-altitude adaptation. Evidence concerning conscientiousness, resilience/hardiness, extraversion, agreeableness, and openness remains more limited and is predominantly indirect or mechanism-informed (Carver & Connor-Smith, 2009; Burtscher et al., 2022).

The following subsections therefore focus on the empirical status of each trait domain, distinguishing directly observed associations from theory-informed interpretations and identifying the exposure phases and boundary conditions under which particular associations may warrant further testing. Evidence from real-altitude field or residential settings, simulated hypoxia, expedition contexts, and adjacent extreme-environment research is interpreted according to its directness rather than treated as interchangeable. Table 3 summarizes the current evidence status, proposed process pathways, relevant boundary conditions, and adaptation-related outcome domains for each trait construct.

### 6.1. Neuroticism, Emotional Stability, and Trait Anxiety

Among the personality-related constructs considered in this review, neuroticism/emotional stability and anxiety-related traits have received comparatively more direct empirical attention than most other trait domains, although the evidence base remains small, heterogeneous, and preliminary. These constructs are conceptually related but should not be treated as interchangeable. Evidence is considered direct support for neuroticism only when neuroticism or a clearly corresponding emotional-stability dimension is explicitly assessed; findings involving trait anxiety, state anxiety, sleep disturbance, or other affective outcomes are interpreted at the level of the construct actually measured.

The most direct early evidence comes from the Everest-Comex 97 experiment, in which eight experienced male climbers underwent a 31-day simulated ascent under hypobaric-chamber conditions. The reports by Nicolas et al. (1999, 2000) were derived from the same highly selected cohort and therefore do not constitute independent replications. Nicolas et al. (1999) reported increasing state anxiety with simulated altitude but found no significant associations between personality traits, including trait anxiety, and altitude symptomatology. Nicolas et al. (2000) subsequently identified an association between greater fatigue and lower 16PF Factor C scores, a dimension related to emotional stability. These findings provide direct but highly limited and context-specific evidence, constrained by the very small sample, restriction to experienced male climbers, extreme simulated exposure, prolonged chamber confinement, and lack of independent replication (Nicolas et al., 1999, 2000). As summarized in Section 5.2, field evidence also indicates that anxiety-related measures are associated with sleep disturbance during early high-altitude exposure; however, such findings should not be interpreted as direct evidence that neuroticism predicts sleep or other high-altitude outcomes.

Taken together, the available literature permits two cautious propositions for further testing. First, anxiety-related characteristics may be associated with selected acute outcomes under high-altitude exposure, but the relevant constructs, exposure settings, and outcome measures vary substantially across studies. Second, neuroticism/emotional stability may represent a broader dispositional vulnerability potentially related to threat appraisal, affective reactivity, and recovery, but this pathway has received only limited direct testing and is motivated partly by broader personality and stress theory (Carver & Connor-Smith, 2009; Lazarus & Folkman, 1984; Burtscher et al., 2022). Neither proposition currently establishes a reliable or generalizable predictive relationship. As discussed in Section 7.3, extrapolation across controlled hypobaric exposure, real-altitude field settings, and prolonged residential exposure should therefore remain cautious.

### 6.2. Conscientiousness

Direct evidence linking conscientiousness to psychological or cognitive adaptation under high-altitude or hypoxic exposure remains limited. Existing studies do not yet establish that conscientiousness reliably predicts sleep recovery, fatigue resistance, adherence to acclimatization behaviors, or cognitive stability. Its role in the present framework is therefore best regarded as a mechanism-informed behavioral-regulation hypothesis rather than an established high-altitude effect.

The theoretical relevance of conscientiousness derives primarily from its broader associations with self-regulation, planning, persistence, and behavioral control (Carver & Connor-Smith, 2009). In high-altitude settings, these characteristics could plausibly influence pacing, recovery behavior, maintenance of sleep–wake routines, fatigue management, and compensatory strategies under sustained demand. Such pathways may be relevant given the marked heterogeneity of cognitive outcomes across exposure conditions, task characteristics, and assessment timing (Bliemsrieder et al., 2022; Fan et al., 2024). However, the hypothesis that conscientiousness contributes particularly to performance stability or sustained functioning, rather than to single-occasion performance, has not been directly tested in high-altitude populations.

The effects of conscientiousness may also depend on workload and recovery opportunity. From a coping and resource-regulation perspective, persistence may support functioning when accompanied by adequate pacing and recovery, but continued effort under insufficient restoration could contribute to cumulative fatigue, sleep disruption, or affective strain (Carver & Connor-Smith, 2009; Hobfoll, 1989). This potentially double-edged pathway remains theoretical in the high-altitude context and requires longitudinal studies that assess conscientiousness, recovery behavior, sleep, fatigue, and performance within the same design (Hobfoll, 1989).

### 6.3. Resilience and Hardiness

Resilience and hardiness are conceptually distinct from the Big Five but remain relevant to the present framework because they emphasize responses to stress and resource preservation. Direct evidence that either construct predicts psychological or cognitive adaptation under high-altitude or hypoxic exposure remains limited. Their proposed role should therefore be regarded primarily as a theory-informed hypothesis concerning longer-term adaptation rather than as an established high-altitude effect.

Their theoretical relevance derives from proposed roles in maintaining engagement under stress, supporting adaptive appraisal and coping, and limiting cumulative resource loss (Carver & Connor-Smith, 2009; Hobfoll, 1989; Kobasa, 1979). Under prolonged high-altitude exposure, these processes may become increasingly relevant as fatigue, repeated stressors, and recovery demands accumulate. Resilience and hardiness may therefore be more informative for understanding trajectories of psychological functioning and recovery than immediate entry-phase symptoms, although these specific pathways remain insufficiently tested in high-altitude populations (Hobfoll, 1989; Kobasa, 1979).

Accordingly, resilience and hardiness are best treated as limited or indirect evidence pathways and as candidates for prospective testing rather than validated predictors of successful adaptation. Longitudinal studies are needed to determine whether they predict psychological stability, fatigue accumulation, recovery, or sustained functioning during prolonged exposure, and whether any such associations operate through appraisal, coping persistence, or preservation of psychological resources (Carver & Connor-Smith, 2009; Hobfoll, 1989; Kobasa, 1979).

### 6.4. Extraversion, Agreeableness, and Openness

Direct evidence linking extraversion, agreeableness, and openness to psychological or cognitive outcomes under high-altitude or hypoxic exposure remains limited. Their proposed relevance is therefore better regarded as limited, indirect, or mechanism-informed, with potential effects operating primarily through social regulation, coping, and responses to environmental novelty rather than through direct modification of hypoxic exposure.

For extraversion, the most plausible pathway is social and affective. Broader personality and coping research links extraversion to social engagement, support seeking, positive affect regulation, and interpersonal communication (Carver & Connor-Smith, 2009). In prolonged high-altitude settings involving group living or coordinated work, these characteristics could facilitate access to social support and emotional recovery, but their value is likely to depend on the availability of social interaction and the broader situational context (Burtscher et al., 2022; Tett & Burnett, 2003; Kristof, 1996). The proposed social-resource pathway should therefore be regarded as context dependent and insufficiently tested in high-altitude populations.

For agreeableness, direct high-altitude evidence is even more limited. Its proposed relevance derives mainly from theories of cooperative coping, interpersonal regulation, and person–environment fit. Under prolonged cohabitation, high workload, or constrained resources, greater agreeableness could plausibly reduce interpersonal conflict and facilitate cooperative support or task coordination (Carver & Connor-Smith, 2009; Kristof, 1996). However, there is currently insufficient evidence that agreeableness predicts psychological stability, team functioning, or cognitive performance specifically under high-altitude hypoxia; any such effects therefore remain socially mediated, mechanism-informed hypotheses requiring direct field testing (Burtscher et al., 2022; Kristof, 1996; Tett & Burnett, 2003).

Openness is likewise best treated as a hypothesis-generating construct. Its broader associations with cognitive flexibility, tolerance of ambiguity, and receptiveness to novel experiences provide a theoretical basis for expecting relevance to unfamiliar routines and environmental demands (John & Srivastava, 1999; Lazarus & Folkman, 1984). Such effects may be more relevant to longer-term adjustment than to acute symptom burden, but this proposition has not been directly established in high-altitude populations. Evidence that cognitive responses vary with exposure duration and task characteristics does not itself demonstrate a specific role for openness (Burtscher et al., 2022; Bliemsrieder et al., 2022; Fan et al., 2024), and direct longitudinal testing is therefore required.

Taken together, the current literature does not establish any personality dimension as a robust predictor of high-altitude adaptation-related outcomes. Neuroticism/emotional stability and anxiety-related traits have comparatively more direct empirical support within the still limited evidence base, particularly for selected acute or early-phase outcomes (Bian et al., 2019; Burtscher et al., 2022; Nicolas et al., 1999, 2000), whereas evidence for conscientiousness, resilience/hardiness, extraversion, agreeableness, and openness remains predominantly limited, indirect, or theory informed (Carver & Connor-Smith, 2009; Hobfoll, 1989; Kristof, 1996; Tett & Burnett, 2003). Personality-related constructs should therefore be viewed as candidate sources of individual variation and targets for prospective testing rather than established determinants of successful or unsuccessful high-altitude adaptation.

## 7. Boundary Conditions and Study Design: Why Personality Effects Are Easily Diluted in High-Altitude Research

Personality-related associations under high-altitude hypoxia are unlikely to be uniform across contexts and may vary with situational demands, exposure time course, task conditions, and sample-selection processes (Carver & Connor-Smith, 2009; Tett & Burnett, 2003). The relevant question is therefore not whether a trait is universally adaptive, but under which conditions and for which outcome domains its association is most likely to become detectable. This issue is particularly important in high-altitude research because hypoxic dose, symptom burden, sleep quality, workload, and social context vary substantially across studies, potentially amplifying, attenuating, or obscuring trait-related associations (Bian et al., 2019; Bloch et al., 2015; Burtscher et al., 2022; Hobfoll, 1989; Kristof, 1996).

### 7.1. Situational Strength and Time Course

Situational strength refers to the extent to which situational cues and demands constrain behavioral variability and thereby limit the expression of dispositional differences. In comparatively strong situations, common situational pressures may produce more uniform responses across individuals, whereas less constrained situations provide greater scope for personality-related differences to become observable (Mischel, 1973; Tett & Burnett, 2003). In the present framework, situational strength is used as a theoretical lens for considering how the expression and detectability of trait-related associations may vary across phases of high-altitude exposure.

Exposure phase is an important boundary condition because the dominant demands of high-altitude environments change over time. During the entry phase, acute bodily sensations, uncertainty, sleep disruption, and rapidly changing symptoms may create a comparatively strong situational context in which immediate affective and symptom-related responses become especially salient. As summarized in Section 5.2, sleep and anxiety-related responses are particularly relevant during this phase. Such acute demands may leave less scope for stable trait differences to be expressed or detected, except where those traits are closely related to threat appraisal or affective reactivity (Bloch et al., 2015). By contrast, during prolonged residence or extended deployment, more stable routines, cumulative workload, recovery demands, and social conditions may provide greater opportunity for individual differences in behavioral regulation, resource management, and social functioning to become observable (Hobfoll, 1989; Kristof, 1996).

Accordingly, associations observed during one exposure phase should not be assumed to generalize to another. Entry-phase studies are better suited to examining acute symptom, sleep, anxiety, and affective responses, whereas longer-term designs are better positioned to evaluate recovery, cumulative fatigue, behavioral regulation, social fit, and maintenance of functioning over time. From a trait–process–outcome perspective, exposure phase should therefore be specified explicitly when interpreting or comparing trait-related findings because the conditions under which personality-related characteristics can be expressed are likely to differ across acute and prolonged exposure (Carver & Connor-Smith, 2009; Hobfoll, 1989; Tett & Burnett, 2003).

### 7.2. Sample-Selection Effects

High-altitude field samples are often not representative of the general population. Participants commonly include mountaineers, expedition members, military or operational personnel, and workers who voluntarily enter demanding environments. Such groups may differ systematically in personality composition before exposure, thereby limiting external validity and reducing trait variability within study samples (Crust, 2020; Monasterio et al., 2014).

This selection can produce range restriction and attenuate observed trait–outcome associations. For example, if a sample is relatively homogeneous in extraversion, conscientiousness, emotional stability, or sensation seeking, limited between-person variability may reduce the statistical detectability of associations with adaptation-related outcomes. Null or weak associations in selected field samples should therefore be interpreted cautiously and considered in relation to the underlying sampling process rather than generalized automatically to broader populations (Crust, 2020; Monasterio et al., 2014).

### 7.3. Exposure-Context Non-Equivalence and Limits of Generalization

Evidence described broadly as “high-altitude” or “hypoxia” research is derived from exposure contexts that are not physiologically, psychologically, or ecologically equivalent. Real-altitude field or residential exposure, hypobaric-chamber simulation, normobaric hypoxia, and mountaineering or expedition settings differ in exposure conditions, duration, workload, recovery opportunity, environmental stressors, and social context. Adjacent extreme-environment studies are less direct because they may reproduce isolation, workload, or resource constraints without reproducing hypoxic exposure itself. The distinction between hypobaric hypoxia (HH) and normobaric hypoxia (NH) is particularly important, as differences in physiological responses and cognitive performance have been reported even under broadly comparable inspired oxygen conditions (Hohenauer et al., 2022). Findings from one exposure paradigm should therefore not be assumed to generalize directly to another.

Real-altitude and controlled-hypoxia studies provide complementary but distinct forms of evidence. Chamber studies permit greater experimental control but do not reproduce the full ecological context of living or working at altitude, whereas field studies provide greater ecological validity while introducing variation in workload, sleep, environmental conditions, prior acclimatization, and social demands. Mountaineering and expedition studies involve additional physical and operational stressors and often rely on self-selected samples whose personality composition may differ from that of broader populations (Crust, 2020; Monasterio et al., 2014). Exposure context should therefore be considered explicitly when evaluating the directness, comparability, and generalizability of trait-related findings.

Temporal generalization requires similar caution. Acute chamber or normobaric hypoxia studies primarily characterize responses over hours or days and cannot, by themselves, establish associations operating during weeks, months, or years of real-altitude residence. Short-duration simulated findings are therefore most appropriately interpreted as evidence for context-specific responses or candidate pathways unless supported by convergent longitudinal or field findings. Cross-study interpretation should additionally consider the adequacy of hypoxic exposure dose characterization, as operationally defined in Section 3.2. Incomplete characterization of exposure dose can increase between-study heterogeneity and limit confidence in comparisons or generalization across exposure paradigms (Hohenauer et al., 2022).

### 7.4. Outcome-Measurement Heterogeneity

High-altitude psychological and cognitive research is characterized by substantial heterogeneity in task paradigms, assessment timing, outcome classification, and control of practice effects (Bliemsrieder et al., 2022; Fan et al., 2024). Meta-analytic evidence further indicates that altitude severity, exposure duration, cognitive domain, and study design contribute to variability in observed effects, making simple classifications of impairment versus non-impairment difficult to interpret across studies (Jiang et al., 2025). Under these conditions, single-occasion mean scores may provide limited sensitivity for detecting person-level differences.

Repeated-measures and trajectory-based designs may therefore be more informative for evaluating trait-related variation in recovery, performance stability, reaction-time variability, or maintenance of functioning under cumulative demand. Such approaches should be regarded as methodological opportunities rather than evidence that personality already predicts these outcomes. Studies relying only on brief assessments or simple pre–post comparisons may miss temporally dynamic associations, particularly when exposure conditions and outcome measures are heterogeneous (Bliemsrieder et al., 2022; Fan et al., 2024; Jiang et al., 2025). Together with the sampling, exposure-phase, and exposure-context issues discussed above, measurement heterogeneity should therefore be considered when interpreting the presence, absence, or generalizability of trait-related associations.

## 8. Future Directions and Methodological Priorities

Future research should move beyond isolated trait–outcome correlations and directly test prespecified trait–process–outcome propositions under clearly characterized exposure conditions. Null or inconsistent findings should be interpreted in light of potential methodological constraints, including exposure heterogeneity, restricted trait variance, limited temporal sensitivity of outcome measures, and inadequate assessment of candidate intermediate processes, while remaining open to the possibility that some trait-related associations are genuinely weak or absent. The priority is therefore not simply to add personality measures to existing high-altitude protocols, but to use longitudinal, process-oriented designs capable of testing when, under what conditions, and through which candidate processes trait-related associations emerge.

### 8.1. Longitudinal and Repeated-Measures Designs

Longitudinal and repeated-measures designs are needed to distinguish entry-phase responses from subsequent stabilization, maintenance, or recovery. Repeated assessment can test whether baseline personality-related characteristics are associated with trajectories of symptom burden, sleep restoration, affective recovery, or cognitive functioning across exposure phases. Compared with single pre–post assessments, such designs are better suited to characterizing temporal dynamics, including recovery rate, within-person fluctuation, and stability of functioning, while also establishing the temporal ordering required to evaluate candidate intermediate processes (Bian et al., 2019; Bliemsrieder et al., 2022; Fan et al., 2024; Jiang et al., 2025).

### 8.2. Exposure Characterization as a Core Design Requirement

Future studies should characterize hypoxic exposure using the multidimensional hypoxic exposure dose framework defined in Section 3.2, rather than relying on nominal altitude alone. Where feasible, reporting should provide sufficient environmental, temporal, and physiological information to characterize both the imposed hypoxic stimulus and the hypoxemia actually experienced by participants. More complete exposure characterization is important for improving cross-study comparability, interpreting variation in psychological and cognitive outcomes, and determining whether apparent between-study differences may reflect variation in exposure conditions rather than person-level factors (Hohenauer et al., 2022; McMorris et al., 2017).

Exposure context should also be specified explicitly, including whether data were obtained during real-altitude field or residential exposure, hypobaric-chamber simulation, normobaric hypoxia, or mountaineering/expedition settings. These paradigms provide complementary but non-equivalent forms of evidence and should not be treated as interchangeable. In particular, findings from acute simulated hypoxia should not be generalized to prolonged real-altitude exposure without convergent longitudinal or field evidence. Clear reporting of both exposure context and hypoxic exposure dose is therefore essential for evaluating the comparability, directness, and generalizability of trait-related findings.

### 8.3. Candidate Intermediate Variables: Sleep, Fatigue, and Affect Regulation

Sleep, fatigue, and affect regulation warrant particular attention in future trait–process–outcome research, but their analytical roles should be specified rather than assumed. Depending on the research question and exposure phase, sleep disturbance and fatigue may function as direct outcomes, correlates or covariates of psychological and cognitive functioning, or candidate intermediate variables linking person-level characteristics to later outcomes. Current evidence supports associations among these domains but does not establish a universal sequential or mediating pathway.

Prospective studies should therefore test mediation explicitly by establishing temporal ordering and evaluating indirect effects within the same design. For example, researchers could examine whether a baseline trait is associated with subsequent sleep disturbance or fatigue and whether these variables, in turn, precede later affective or cognitive outcomes. Until such temporal and statistical evidence is available, sleep, fatigue, and affect-regulation variables should be described as candidate intermediate processes or potential mediators rather than established mechanisms (Bian et al., 2019; Bloch et al., 2015; Burtscher et al., 2022).

### 8.4. Ecological Validity and Interpretive Structure

Future research should better align study design with the environmental and operational conditions under which high-altitude outcomes occur. In addition to hypoxic exposure, workload, group living, role demands, recovery opportunity, and social support may contribute to variability in psychological and cognitive functioning. Laboratory studies remain valuable for experimental control, whereas field-based or hybrid designs may be better suited to examining whether trait-related associations vary with real-world task and social demands (Kristof, 1996; Tett & Burnett, 2003).

Greater ecological validity, however, should not come at the expense of interpretability. Studies should clearly specify exposure context and phase, outcome domain, relevant contextual demands, and key person-level covariates so that trait-related associations can be distinguished from environmental or design-related sources of variation. Such designs would allow ecologically meaningful hypotheses to be tested without treating context-dependent or theory-informed pathways as established effects.

### 8.5. A Proposed Measurement Agenda for Future Trait-Based High-Altitude Research

To facilitate direct testing of trait–process–outcome hypotheses, future studies may benefit from a theory-aligned measurement strategy covering dispositional characteristics, exposure, candidate processes, and domain-specific outcomes. This section is intended as a proposed measurement agenda rather than an evidence-based minimum assessment battery. The instruments listed below are illustrative examples and were not selected through a formal comparison of psychometric performance, feasibility, cultural validity, respondent burden, or suitability for high-altitude environments.

At the dispositional level, measurement should correspond closely to the construct specified in the hypothesis. Broad personality traits may, for example, be assessed within the Big Five framework using instruments such as the BFI-2, whereas narrower constructs may require measures of trait anxiety, coping-related tendencies, resilience, or hardiness (Soto & John, 2017; Spielberger et al., 1983; Carver, 1997; Connor & Davidson, 2003). Instrument selection should consider reliability, validity, linguistic and cultural adaptation, and measurement equivalence in the target population rather than assuming that a commonly used measure is automatically suitable for a particular high-altitude sample. Exposure variables should likewise be selected in accordance with the exposure context and phase, following the characterization principles outlined in Section 8.2.

Measurement of candidate intermediate variables should be pathway specific rather than standardized across all studies. For hypotheses involving sleep or fatigue, illustrative measures include the Pittsburgh Sleep Quality Index, Insomnia Severity Index, Karolinska Sleepiness Scale, and repeated vigilance-sensitive paradigms such as the Psychomotor Vigilance Test (Åkerstedt & Gillberg, 1990; Bastien et al., 2001; Buysse et al., 1989; Dinges & Powell, 1985). Affect-related variables may be assessed using instruments such as the PANAS, GAD-7, or PHQ-9 when appropriate to the hypothesis and exposure phase (Kroenke et al., 2001; Spitzer et al., 2006; Watson et al., 1988). As emphasized in Section 8.3, sleep, fatigue, and affective variables should be treated as candidate intermediate variables or outcomes according to the study design rather than assumed to be established mediators.

Outcome measurement should remain domain specific. Cognitive measures should be selected according to the process of interest and may include indices of sustained attention, executive control, reaction-time variability, or performance stability. Brief vigilance paradigms and workload-sensitive measures such as the NASA Task Load Index may be useful in particular designs but are not proposed as universally preferred measures (Dinges & Powell, 1985; Hart & Staveland, 1988). Where social or ecological functioning is relevant, documentation of isolation, confinement, social context, or operational demands may also be informative, particularly in field or expedition settings (Golden et al., 2018; Goerke et al., 2024; Palinkas et al., 2000; Palinkas & Suedfeld, 2021). Acute symptom- or anxiety-related measures may likewise be included when relevant to the study question and exposure phase (Tang et al., 2023; Weil, 2004).

Overall, the proposed agenda prioritizes measurement alignment rather than instrument standardization. Selection should be guided by the prespecified hypothesis, target population, exposure context and phase, cultural and linguistic validity, repeated-measure requirements, respondent burden, and field feasibility. Measures validated primarily in low-altitude or general populations should not be assumed to retain identical psychometric or practical properties under high-altitude conditions. Comparative methodological research examining psychometric performance, cultural equivalence, repeated-measure sensitivity, and field feasibility is therefore needed before a standardized high-altitude assessment battery can be justified.

## 9. Limitations

Several limitations should be considered when interpreting this review. First, this study was conducted as a structured narrative review rather than an exhaustive systematic review. Although the search and eligibility procedures were made explicit, the search was not designed to capture every potentially relevant publication. Source selection followed an evidence-prioritization approach and was supplemented by citation tracing, which inevitably retains some degree of reviewer judgment. In addition, no formal study-level risk-of-bias assessment or certainty-of-evidence grading was conducted. The evidence categories used throughout the review should therefore be interpreted as qualitative indicators of relative support rather than formal evaluations of methodological quality or evidential certainty.

Second, limitations of the underlying literature constrain the proposed trait–process–outcome framework. Direct high-altitude or controlled-hypoxia evidence remains sparse for several personality-related constructs, and many proposed pathways rely partly on broader personality, stress, coping, extreme-environment, or person–environment research. Considerable heterogeneity also exists in exposure context and duration, personality and related constructs, psychological and cognitive outcome definitions, and measurement procedures. Findings obtained under real-altitude residence, hypobaric chambers, normobaric hypoxia, or expedition settings are therefore not directly interchangeable, and the available evidence is insufficient to establish a single generalizable model across exposure conditions or outcome domains.

Third, separating stable trait-related variation from concurrent psychological states and contextual influences remains difficult. Constructs such as neuroticism and trait anxiety overlap conceptually with, but are not equivalent to, state anxiety, affective distress, symptom reporting, sleep disturbance, or fatigue. At the same time, workload, prior acclimatization, social context, self-selection into high-altitude environments, and other environmental or person-level factors may influence both measured traits and adaptation-related outcomes. Most existing studies do not provide the longitudinal, repeated-measures, or multivariable designs required to disentangle these influences or establish temporal mediation. Accordingly, the pathways proposed in this review should be regarded as hypothesis-generating and differentially supported research targets rather than established causal mechanisms or validated predictors of high-altitude adaptation.

## 10. Conclusions

High-altitude hypoxia is associated with heterogeneous psychological and cognitive outcomes whose expression varies across exposure phase, outcome domain, and study context. Sleep disturbance is among the more consistently observed early outcomes, whereas affective and cognitive effects are more variable and depend substantially on exposure characteristics, duration, and measurement approach (Bloch et al., 2015; Bliemsrieder et al., 2022; Fan et al., 2024; Jiang et al., 2025).

Within this heterogeneous evidence base, no personality-related construct can currently be regarded as an established predictor of high-altitude outcomes. Anxiety-related characteristics and neuroticism/emotional stability have received comparatively more direct investigation than most other trait domains, whereas proposed pathways involving conscientiousness, resilience/hardiness, extraversion, agreeableness, and openness remain more limited, indirect, or mechanism-informed.

## Figures and Tables

**Table 1:** Database-specific search strategies used in the structured narrative review.

Database/Module	Complete Search Strategy	Retrieval
PubMed–Search A	(“high altitude”[Title/Abstract] OR “high-altitude”[Title/Abstract] OR “hypobaric hypoxia”[Title/Abstract] OR “normobaric hypoxia”[Title/Abstract] OR “simulated altitude”[Title/Abstract]) AND (sleep[Title/Abstract] OR fatigue[Title/Abstract] OR anxiety[Title/Abstract] OR depression[Title/Abstract] OR mood[Title/Abstract] OR cognition[Title/Abstract] OR cognitive[Title/Abstract] OR attention[Title/Abstract] OR “executive function”[Title/Abstract] OR memory[Title/Abstract]) AND (“systematic review”[Title/Abstract] OR “meta-analysis”[Title/Abstract] OR “meta analysis”[Title/Abstract] OR review[Publication Type]) AND English[Language]	304
PubMed–Search B	(“high altitude”[Title/Abstract] OR “high-altitude”[Title/Abstract] OR “hypobaric hypoxia”[Title/Abstract] OR “simulated altitude”[Title/Abstract]) AND (personality[Title/Abstract] OR neuroticism[Title/Abstract] OR extraversion[Title/Abstract] OR conscientiousness[Title/Abstract] OR agreeableness[Title/Abstract] OR openness[Title/Abstract] OR “trait anxiety”[Title/Abstract] OR resilience[Title/Abstract] OR hardiness[Title/Abstract] OR coping[Title/Abstract] OR “sensation seeking”[Title/Abstract]) AND (adapt*[Title/Abstract] OR symptom*[Title/Abstract] OR “acute mountain sickness”[Title/Abstract] OR sleep[Title/Abstract] OR fatigue[Title/Abstract] OR anxiety[Title/Abstract] OR mood[Title/Abstract] OR depression[Title/Abstract] OR stress[Title/Abstract] OR cognit*[Title/Abstract] OR attention[Title/Abstract] OR memory[Title/Abstract] OR performance[Title/Abstract]) AND English[Language]	212
Web of Science–Search A	Topic = (“high altitude” OR “high-altitude” OR “hypobaric hypoxia” OR “normobaric hypoxia” OR “simulated altitude”) AND (sleep OR fatigue OR anxiety OR depression OR mood OR cognition OR cognitive OR attention OR “executive function” OR memory) AND (“systematic review” OR “meta-analysis” OR “meta analysis” OR review); Language = English	307
Web of Science–Search B	Topic = (“high altitude” OR “high-altitude” OR “hypobaric hypoxia” OR “simulated altitude”) AND (personality OR neuroticism OR extraversion OR conscientiousness OR agreeableness OR openness OR “trait anxiety” OR resilience OR hardiness OR coping OR “sensation seeking”) AND (adapt* OR symptom* OR “acute mountain sickness” OR sleep OR fatigue OR anxiety OR mood OR depression OR stress OR cognit* OR attention OR memory OR performance); Language = English	489

**Table 2:** Operational criteria for qualitative evidence tiering.

Evidence Category	Operational Criteria	Typical Interpretation
Stronger/convergent evidence	Multiple directly relevant high-altitude/hypoxia studies and/or review-level evidence; independent replication; generally consistent findings; appropriate and validated measurements	Association/pathway has comparatively robust direct support
Moderate/emerging direct evidence	At least one direct high-altitude/hypoxia study or a small set of convergent direct studies; limited replication or design/measurement coverage	Directly supported but not yet sufficiently replicated
Limited/indirect evidence	Sparse, inconsistent, or methodologically limited direct evidence; substantial reliance on adjacent extreme-environment or indirect process evidence	Plausible but weakly established in high-altitude settings
Mechanism-informed hypothesis	Primarily supported by established theory or evidence outside the high-altitude context; little or no direct testing of the proposed trait–adaptation pathway	Testable hypothesis rather than established empirical association

**Table 3:** Evidence status and plausible pathways for personality-related traits in high-altitude adaptation.

Trait Domain	Main Process Pathway	Exposure Phase/Boundary Conditions	Adaptation Outcomes Most Likely Affected	Direct High-Altitude/Hypoxia Evidence	Theoretical/Mechanism-Informed Support	Overall Evidence Status
Neuroticism/emotional stability	Heightened threat appraisal, interoceptive monitoring, negative interpretation bias, and affective arousal.	Most salient during entry or acute exposure, especially when symptoms are ambiguous and sleep is disrupted.	Subjective symptom burden, anxiety, sleep disturbance, affective vulnerability, and early maladaptation.	Nicolas et al. (1999, 2000): same Everest-Comex 97 cohort (*n* = 8); direct but mixed and highly context-specific evidence. Bian et al. (2019) provides anxiety/sleep evidence but not direct evidence for neuroticism.	Burtscher et al. (2022) and broader personality/stress interpretation discussed in Section 6.1.	Limited and mixed direct evidence; comparatively more directly investigated than most other trait domains, but not independently replicated.
Trait anxiety/anxiety sensitivity	Proximal vulnerability to threat appraisal, bodily sensation amplification, anticipatory worry, and disturbed recovery.	Particularly relevant in early ascent, acute exposure, or unfamiliar hypoxic settings with high uncertainty.	Anxiety-linked sleep disturbance, AMS-related symptom reporting, fatigue, and affective instability.	Nicolas et al. (1999, 2000) and anxiety-related field findings from Bian et al. (2019) and Tang et al. (2023); measured anxiety constructs are not necessarily interchangeable.	Broader threat-appraisal and vulnerability interpretation discussed in Section 6.1.	Limited direct evidence; construct and outcome heterogeneity preclude established predictive claims.
Conscientiousness	Behavioral self-regulation, adherence to pacing and recovery routines, effort control, and risk management.	More likely to matter during prolonged exposure, structured work, sustained task demands, or limited recovery opportunity.	Fatigue management, performance stability, error-rate control, adherence, and sustained functioning.	No clear direct high-altitude evidence identified in the cited literature.	Self-regulation, coping, and resource-management literature (Carver & Connor-Smith, 2009; Hobfoll, 1989).	Mechanism-informed hypothesis.
Resilience/hardiness	Resource preservation, adaptive appraisal, coping persistence, motivation maintenance, and recovery after repeated stressors.	Most relevant to mid- or long-term residence, deployment, or repeated exposure rather than immediate symptom prevention.	Longer-term psychological stability, cumulative fatigue resistance, recovery, and maintenance of functioning.	Direct longitudinal high-altitude evidence remains very limited.	Stress-resilience and resource-preservation theory (Hobfoll, 1989; Kobasa, 1979).	Limited/indirect evidence; primarily theory informed.
Extraversion	Social support seeking, positive affect regulation, communication, and team engagement.	Context-dependent: potentially beneficial in group settings but less protective under isolation, monotony, or restricted social contact.	Social adjustment, emotional recovery, perceived support, and distress under social deprivation.	Direct trait-specific high-altitude evidence remains limited.	Review-level, person–environment, and trait-activation perspectives (Burtscher et al., 2022; Kristof, 1996; Tett & Burnett, 2003).	Limited/indirect and context-dependent evidence.
Agreeableness	Conflict reduction, cooperative coping, interpersonal regulation, and redistribution of social or task demands.	More likely to emerge during prolonged cohabitation, team operations, cumulative fatigue, or resource constraints.	Team functioning, interpersonal stress, psychological safety, and indirectly affective or cognitive stability.	No clear direct high-altitude evidence identified in the cited literature.	Cooperative coping, person–environment fit, and adjacent extreme-environment evidence (Carver & Connor-Smith, 2009; Golden et al., 2018; Kristof, 1996).	Mechanism-informed hypothesis.
Openness	Cognitive flexibility, tolerance of ambiguity, novelty adaptation, and meaning making.	More relevant to adaptation trajectories, unfamiliar routines, and environmental novelty than to acute symptom burden.	Adjustment to new routines, psychological persistence, flexible coping, and potentially cognitive adaptation trajectories.	No clear direct high-altitude evidence identified in the cited literature.	Broad personality and appraisal/flexibility theory (John & Srivastava, 1999; Lazarus & Folkman, 1984).	Mechanism-informed hypothesis.

## Data Availability

The data and materials generated during and/or analyzed during the current study are available from the corresponding author on reasonable request.
